# Fluid Gels: a New Feedstock for High Viscosity Jetting

**DOI:** 10.1007/s11483-018-9523-x

**Published:** 2018-03-16

**Authors:** Sonia Holland, Chris Tuck, Tim Foster

**Affiliations:** 1 Division of Food Science, Sutton Bonington Campus, University of Nottingham, Loughborough, LE12 5RD UK; 2 3D Printing and Additive Manufacturing Research Group, University Park, University of Nottingham, Nottingham, NG7 2RD UK

**Keywords:** 3D printing, Agar, Fluid gel

## Abstract

Suspensions of gel particles which are pourable or spoonable at room temperature can be created by shearing a gelling biopolymer through its gelation (thermal or ion mediated) rather than allowing quiescent cooling – thus the term ‘fluid gel’ may be used to describe the resulting material. As agar gelation is thermoreversible this type of fluid gel is able to be heated again to melt agar gel particles to varying degrees then re-form a network quiescently upon cooling, whose strength depends on the temperature of re-heating, determining the amount of agar solubilised and subsequently able to partake in re-gelation. Using this principle, for the first time fluid gels have been applied to a high viscosity 3D printing process wherein the printing temperature (at the nozzle) is controllable. This allows the use of ambient temperature feedstocks and by altering the nozzle temperature, the internal nature (presence or absence of gel particles) and gel strength of printed droplets differs. If the nozzle prints at different temperatures for each layer a structure with modulated texture could be created.

## Introduction

The term fluid gel describes the suspension of microgel particles created when sufficient shear is applied to a gelling biopolymer solution through its gelation, as opposed to allowing the solution to gel quiescently. This has been described in the literature for thermoreversible biopolymer solutions [1–11], including mixed systems [12–14], but also biopolymers which undergo gelation mediated to some extent by ions [15–17]. Factors influencing fluid gel production include the shearing method (Couette or cone and plate geometries in a rheometer, jacketed pin stirrer, overhead impeller or magnetic flea), shear rate, cooling rate, biopolymer type and concentration and ion concentration, if required. These variables will influence the size, shape and number of fluid gel particles created and therefore their measureable properties, crucially their rheological [8] and thermal [4] behaviour. Anisotropic, sometimes ‘hairy’, fluid gel particles have been found in a number of studies [3, 4, 11, 13] this is in contrast with other studies where very small, spherical fluid gel particles were produced [13, 15, 16]. An early patent in the area [2] states that, ideally, microgel particles should not exceed 200 μm in diameter, but more preferably be under 100 μm with a large proportion having a diameter less than 50 μm.

A number of studies discuss the possible mechanism of formation of these fluid gel particles, there is general agreement in the literature that fluid gels arise as a result of shear induced phase separation, creating polymer-rich and polymer-poor regions within a solution undergoing gelation due to either spinodal decomposition or a nucleation and growth mechanism. This demixing through gelation in shear constrains gelation and potentially influences the kinetics of network formation inside the gel particle forming a suspension of discrete gel particles in a predominantly aqueous continuous phase. The initially formed gel nuclei grow to an equilibrium particle size, determined by the shear field experienced [4, 8, 9, 11, 16, 18].

Agar is one such biopolymer, extracted from red seaweed, which will undergo thermoreversible gelation. The agar must be dispersed in solvent (typically water) then heated and held at high temperature (usually 90–100 °C) to ensure polymer chain solubilisation, after which the chains adopt a random coil configuration. Upon cooling, single agar chains form double helices which are then able to aggregate further to create the gel state, as described by the “Domain Model” [19, 20]. Agar is able to adopt this ordered confirmation due to the regular repetition of the disaccharide building block, comprised of alternate 1,3 linked β-D-galactopyranose and 1,4 linked 3,6-anhydro-α-L-galactopyranose. In actual fact agar has two components; agarose, which is linear, neutral and able to gel and a small amount of non-gelling agaropectin which is slightly acidic due to the presence of glucuronic or pyruvic acid residues and sulphate ester. Substantial hysteresis is seen between the helix to coil transition on heating and coil to helix upon cooling. This hysteresis is apparent due to helix stabilisation within aggregates, thus the helix to coil transition occurs at a higher temperature and is typically broader when observed via thermal analysis than the coil to helix transition [21, 22]. Early studies determined that fluid gel systems exhibit gel-like rheological responses, suggesting that gel particles themselves have similar strength to their quiescent counterparts at given concentrations; thus lower concentrations lead to softer, more deformable particles and vice versa [8].

Intended application of fluid gels has arisen from their colloidal nature, giving potential as fat replacers in foods or for use in personal care products. There has been one instance of application in 3D printing whereby the interaction between fluid gel particles in suspension has been utilised as a ‘self-healing’ matrix substrate for a technique known as suspended manufacture [23]. In this technique the agarose fluid gel is used as a suspending agent to allow the production of scaffold structures from a variety of hydrogels extruded from a syringe above the fluid gel bed. These hydrogel structures could then be cross linked by addition of CaCl_2_ and removed from the fluid gel supporting medium once gelation had occurred. 3D printing applications using gelling agents as the main material typically utilise this material extrusion method, often referred to as Filament Freeform Fabrication (FFF) or Fused Deposition Modelling (FDM) [24]. For biomedical application it is common to use a gelling polymer and further strengthen the structure through modifications to allow photo crosslinking on exposure to UV light, such as methacrylation of monomer building blocks and addition of a photoinitiator [25]. Care must be taken when using these systems as the UV light splits the photoinitiator molecule to form free radicals. These free radicals are able to react with proteins and other biological components which could negatively impact on the intended function of the structure, for example if live cells are included in the ink for biomedical application. In addition, photoinitiators are generally toxic, so this type of crosslinking modification would not be suitable for food applications [26, 27].

One study by Gilon et al. [28] describes the use of gelatine methacrylate (GelMA) in a high viscosity jetting technique. Unlike conventional ink jet where ink viscosity, surface tension and density must be tightly controlled to achieve optimum jetting characteristics [29] this microvalve system is able to dispense higher viscosity inks through piezo actuated rod movement determining the opening and closing of a valve. These systems also deal well with non-Newtonian inks as described by Ledesma-Fernandez et al. [30] however, bio-applications with live cells suggest clogging of nozzles can be an issue despite microvalve nozzle diameters being larger than conventional ink jet, in the range of 50-150 μm [27]. Using agar fluid gels as a feed stock for 3D printing using a microvalve system enables the thermal gelation to be exploited in order to create structures of varying hardness by heating and cooling the nozzle during printing. A temperature dependent partial or full melting of fluid gel particles can be induced; re-ordering of agar chains on quiescent cooling based on the degree of melting out that has occurred solidifies the resulting structure. For a food application the extent to which re-ordering can take place will create layers with varying gel strength, there is also potential for application in the biomedical sector by incorporating cells into the agar fluid gel particles prior to printing.

## Materials and Methods

Two agars were used to create fluid gels: Luxara-1253 (Arthur Branwell & Co Ltd., UK) whose composition is available in [5], also used to create fluid gels in [4, 7, 8, and], and agar-agar for microbiology (Sigma Aldrich, UK) with manufacturer T_gel_ = 35 °C and 1.5% gel solubility in water at 75 °C.

A 1% stock solution of sodium azide (Sigma Aldrich UK) was diluted to 0.1% in fluid gel formulations.

### Formation of Fluid Gels

#### Stirred Pot

A jacketed vessel with a magnetic stirrer was used to create larger volumes of fluid gels. The vessel was filled with 20 g ultrapure water (with 0.1% NaN_3_) for 2% solutions and 15 g water for 3% solutions then 0.4 or 0.45 g, respectfully, of agar was gently added whilst agitating. The vessel was attached to a water bath which was heated to 90 °C and held at this temperature for 30 min to fully solubilise the agar. Whilst stirring at 1000 rpm the water bath cooled the solution to 10 °C at a rate of 1.5°Cmin^−1^, determined using a temperature probe. For continuity throughout experiments this was the heating and cooling rate carried forward for rheology. Fluid gels were stored at 4 °C until use.

#### Rheometer

Creation of fluid gels and rheological tests were conducted on the MCR 301 rheometer (Anton Paar, Austria) using a cone and plate geometry (50 mm diameter with 50 μm truncation and 2° cone angle) with TruGap functionality and a moisture trap to prevent drying of the sample during heating and cooling stages. Agar solutions were prepared at 2 or 3% via the cold dispersion method described above and a minimum of 30 min stirring at 90 °C was allowed for polymer solubilisation. 1500 μL of hot solution was pipetted into the centre of the plate at 85 °C. Fluid gels were created in the rheometer through application of constant shear at 400 s^−1^ whilst cooling at 1.5°Cmin^−1^ to 15 °C. These gels could be collected for microscopy or remain in the rheometer for further analysis. Amplitude sweeps showed the linear viscoelastic (LVE) range to be between 0.01–1% strain (results not shown). Oscillatory temperature sweeps were conducted within the LVE range (0.05%) at angular frequency (ω) = 10rads^−1^ to heat and cool the fluid gels at 1.5°Cmin^−1^ to temperatures between 55 and 85 °C whilst measuring the elastic and viscous moduli (*G’* and *G”* respectively). Fluid gels made via the stirred pot method were also subject to amplitude sweeps and the heating and cooling regimes.

### Phase Contrast Microscopy

An Axiovert 200 M Wide Field Fluorescence Microscope (Zeiss, UK) was used to conduct phase contrast microscopy with ×10, ×40 and ×63 objectives.

### Centrifugation

Fluid gel samples were centrifuged using a Jouan CR3i (Thermo Electron Corporation, UK) at 4100 rpm/2500G for 30 min at a set temperature of 10 °C. The supernatant and pellet portions of each sample were separated and weighed to determine the effective phase volume of fluid gel particles versus the continuous phase.

### Micro DSC

A Setaram MicroDSC III calorimeter (SETARAM, France) was used to observe ordering and disordering transitions on application of thermal energy. With water as a reference, ≈800 mg of fluid gel sample was loaded into the sample cell at 20 °C. Samples were heated to varying temperatures between 55 and 100 °C and cooled to 10 °C, following this was an immediate reheating step to assess the resulting gel sample; the rate used remained 1°Cmin^−1^. Enthalpies of transition are quoted as Jg^−1^ of agar.

### High Viscosity Jetting

A 3-Dimensional stage, controlled by a computer, was used to determine the position of a PICO®Pμlse™ (Nordson EFD) jetting valve throughout printing with overall accuracy of 5 μm; nozzle sizes used were 100 and 150 μm, as the available 50 μm was deemed too close to particle size and risked blockage during printing runs. Cycle time was constant at 10 ms, relating to a frequency of 100 Hz, however stroke, voltage, pulse, open and close times were varied using a PICO Toμch controller and a locking air pressure regulator used to deliver the required pressure.

### Light Microscopy

An Eclipse LV100ND (Nikon UK Ltd.) was used to visualise printed structures as soon after printing as possible, to avoid the potential effects of drying.

## Results and Discussion

### Particle Morphology and Phase Volume

**Fig. 1:**
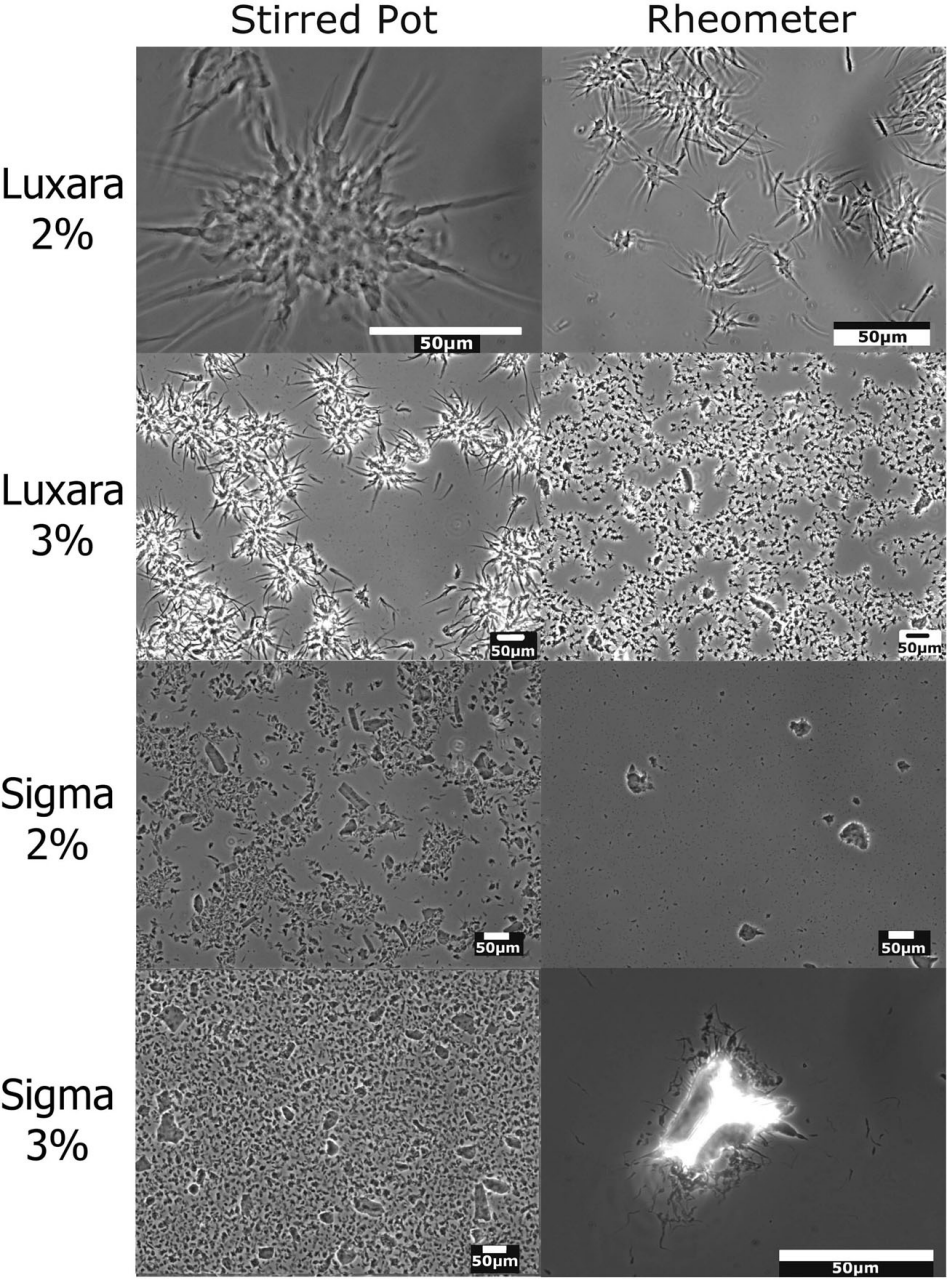
Phase contrast micrographs of fluid gels. Left column; stirred pot, Right column; rheometer. Rows 1 and 2; Luxara agar, 2 and 3% (respectively). Rows 3 and 4; Sigma agar, 2 and 3% (respectively)

As mentioned in the introduction discrepancies exist in the literature as to the size and shape of fluid gel particles produced, even from agar of the same source at equal concentrations. Figure 1 shows phase contrast micrographs of fluid gel particles produced in this work, of two agars at two concentrations produced via two different processes. The Luxara agar tended to form particles with a semi-spherical core and many protruding, long ‘tails’ into the continuous phase; these ‘tails’ give the particles a much larger hydrodynamic volume than if only the core portion was present. Authors in previous work [4, 7, 11] have also noted the formation of these ‘tails’, however consensus has previously been that after initial particle formation there is a period of further molecular ordering, wherein these tail structures re-form with the main particle core over time to produce a smoother particle with a more spherical shape determined by the flow field. From these micrographs it is evident that this is not the case in this instance and similar observations were made by Ellis et al. [11] where their explanation again states the need for controlled flow fields [16]. In our study fluid gel particles produced using agar-agar from Sigma Aldrich also have these tails but they do not protrude as far into the continuous phase as tails on Luxara gel particles. Therefore, the effect on hydrodynamic volume is less pronounced for the Sigma agar particles and, as a result, these particles appear smoother.

Here the size and shape of all fluid gel particles was variable throughout the different production processes and concentrations investigated. Aside from the presence or absence of ‘tails’ the particle core ranged from near spherical in shape to anisotropic, often with a high aspect ratio (as seen particularly with the Sigma agar fluid gels, produced in the stirred pot at 2% concentration, and Luxara fluid gels produced in a rheometer at 3% concentration, respectively). A trend for the size of particles to decrease as the concentration of agar in solution increased was noted which is consistent with findings in the literature [4, 16, 18], as well as when a rheometer cone and plate flow field was used rather than the stirred pot method. These visual observations can help explain fluid gel behaviour in further analysis, below.

Samples for phase contrast microscopy were diluted to allow better visualisation of individual particles, therefore little information can be gathered from these regarding particle numbers versus continuous phase volume. After 30 min of centrifugation the supernatant of 2% fluid gels made with Sigma agar was found to be a smaller volume than those made with Luxara agar, thus the pellet collected for Sigma comprised a larger phase volume of the original sample than the Luxara. Based on the weight of separated supernatant and pellet samples collected the Sigma fluid gel particles contributed to 85 ± 6% phase volume whereas Luxara particles only comprised 59 ± 2% phase volume of the total sample. Therefore we can conclude that the effective volume of the tails present will have an effect and result in different behaviour exhibited by the Luxara and Sigma fluid gels.

### Thermal Behaviour of Fluid Gels

Norton et al. [4] previously identified the link between a DSC thermogram of an agar fluid gel versus the increase in viscosity due to the formation of double helices and subsequent aggregation. It was this information which provided the first explanation of the mechanism by which fluid gels are created. They also show that through different treatment of agarose samples (such as heating and cooling or conducting isothermal holds to cure samples over a number of days) that the melting profile as depicted by a DSC thermogram may vary. This observation was also noted by Aymard et al. [31] through varying isothermal hold temperatures close to and away from the gelation temperature.

These observations from the literature on the effect of heating and cooling on the extent of ordering can be used to explain observations presented here based on DSC thermograms.

**Fig. 2:**
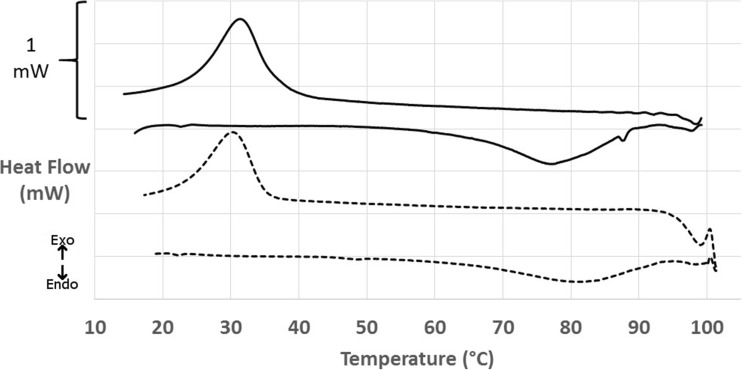
MicroDSC showing the thermal transitions of Luxara (solid) and Sigma (dashed) agar fluid gels heated to 100 °C and cooled at 1°Cmin^−1^

Figure 2 shows full melting and cooling profiles of 2% agar fluid gels, heated to 100 °C to ensure aggregates were fully destabilised and helices had completed their transition to become single coils in solution. Thermograms displayed the same trends for thermal hysteresis of the transitions on heating and cooling and a broader transition during heating versus a narrower transition observed on cooling as described in the literature [21, 22]. It is interesting to note that the entire Sigma agar endothermic transition is shifted a few degrees higher than the Luxara, whereas the exothermic transition is 1 °C lower, with both transitions being broader for Luxara than Sigma. When the integral of transition peaks are plotted with respect to the enthalpy of the transition the extent of completion, or ordering/disordering, for a given temperature point can be deduced. The peak maximum of the Luxara melting endotherm was 76.8 ± 0.9 °C, but at this point only 55% of the transition is complete. The same methodology can be used for the exothermic transition; for Sigma agar the exothermic peak maximum was 30.0 ± 0.1 °C yet at this point the transition was only 41% complete, this will be discussed further with respect to fluid gel rheological responses.

**Fig. 3:**
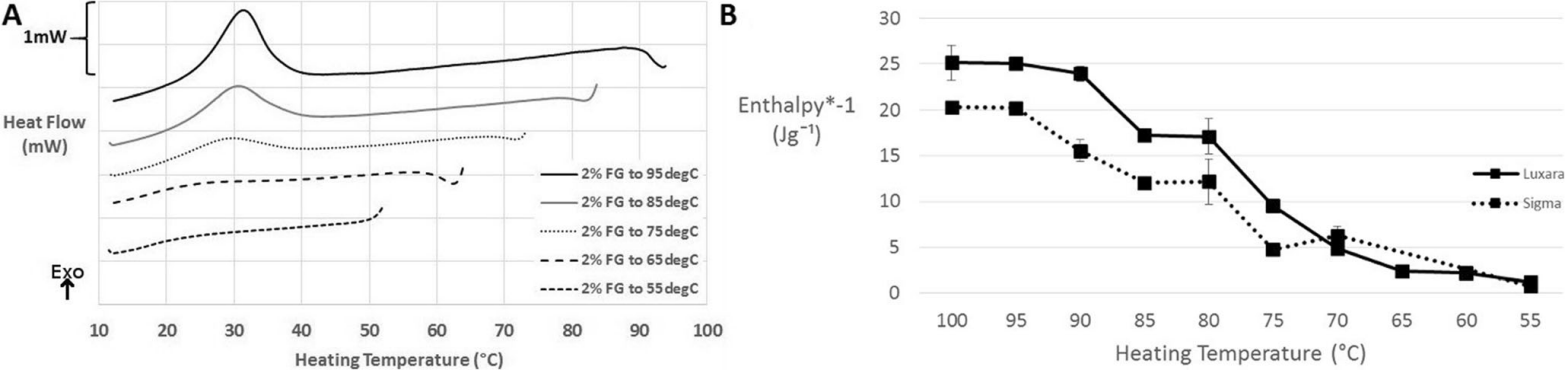
**a** Exotherms obtained after selectively heating Luxara fluid gels to 55–95 °C and cooling. **b** A plot of the enthalpy of exotherms versus the temperatures to which the samples were heated (Luxara and Sigma)

For the purposes of applying fluid gels to a temperature controlled high viscosity print head, in order to use thermal energy input to gain printed gel droplets of varied hardness, it was interesting to observe the extent of re-ordering depicted by the gelation exotherm after an initial heat to a defined temperature (between 55 and 100 °C). Figure 3 shows that there was a distinct trend of a reduction in the area of these exotherms the lower the temperature to which the fluid gel was heated to. Graph B of Fig. 3 shows the calculated enthalpies (converted to positive values for the purposes of graphical representation) versus the heating temperature. Enthalpy values obtained upon full melting for both agars (i.e. heating to 95–100 °C) are concordant with the literature value of 23 ± 3 Jg^−1^ obtained by Cooke et al. [21] and Gidley et al. [32]. As the temperature to which the samples were heated to decreased the enthalpy obtained upon gelation decreased, which can provide insight into the ordering fluid gel particles underwent during formation. Depending on the temperature, aggregates will be destabilised and helices more or less solubilised in single coil state, thus able to partake in gelation upon the cooling, influencing the enthalpy of obtained exothermic peaks. As expected the samples heated to temperatures below the melting peak maximum exhibit much lower enthalpies, and the heat supplied may only just be enough to break aggregates but not effect helical transition. Of particular interest is the gelation behaviour after heating to temperatures between the peak maximum of the melting endotherm and the endset of transition. This is due to the emergence of a plateau region after heating to both 80 °C and 85 °C, which will be discussed further when linked to a rheological phenomenon in the next section.

### Rheological Studies of Fluid Gels over Temperature

**Fig. 4:**
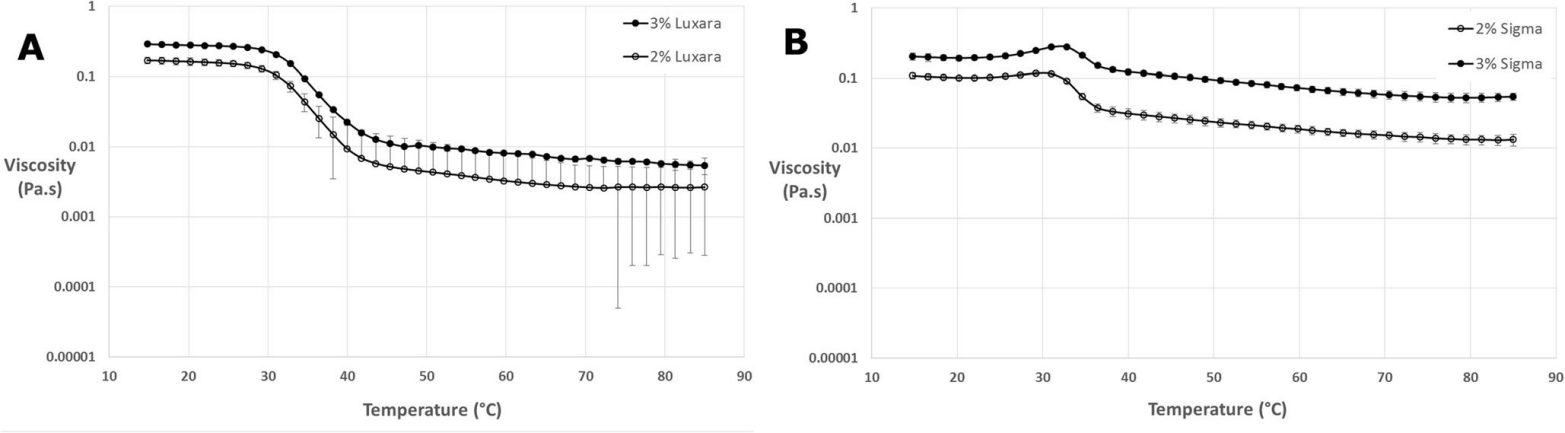
Flow curves of fluid gel formation in a rheometer at 400 s^−1^ for 2 and 3% Luxara (**a**) and Sigma (**b**) agar

For fluid gels created in the rheometer (at 400 s^−1^) the viscosity was measured throughout and can be plotted against temperature as shown in Fig. 4 to observe changes occurring during this process. Graph A depicts formation of gels with Luxara agar whereas graph B shows Sigma agar fluid gel formation. As expected, the final viscosity for the 3% dispersions is higher than for the 2% dispersions. The temperature range during which the viscosity increases most rapidly coincides with the gelation exotherm determined by μDSC for both types of agar. It is interesting to note the appearance of a peak after the rapid rise in viscosity for fluid gels produced only with Sigma agar and not with those produced using Luxara which occurs at 29.9 ± 0.9 °C, coinciding with the exothermic peak maximum obtained from μDSC. By 25 °C the viscosity begins to reach an equilibrium. When considering results obtained through integration of the endothermic transition peak integrated it is apparent that 40% of the transition occurs within this temperature region (25–30 °C). The appearance of this peak is also noted in Norton et al. [4] but its disappearance corresponds to lowering concentration (not evident at 1% but is at 2 and 3%). Peaks such as this are expected in the presence of close packed particles and Norton et al. [4] conclude that at the lowered concentration of 1% there are fewer gelling nuclei in the matrix for fluid gel particle formation and lower local shear stresses experienced by the particles, leading to a lower volume fraction of overall larger particles thus the peak disappears. On centrifugation of Sigma and Luxara fluid gel samples (described above in the Particle Morphology and Phase Volume section) the volume fraction of particles versus continuous phase was found to be much less for Luxara gel particles than Sigma ones, therefore in the Luxara sample particles would not exhibit a ‘jamming’ phenomenon on formation as seen in the Sigma sample. Additionally, the protruding ‘tails’ on the Luxara fluid gel particles may enable overlap and ease of movement of particles in shear during the latter stages of ordering upon cooling, since this lower temperature state is the likely origin of tail formation after the particle core. Norton et al. [4] also discuss the decrease in viscosity being a result of a decrease in hydrodynamic volume of particles, upon subsequent ordering after all particles have been nucleated/formed. The formation of heterogenous particles for Luxara clearly indicates that such a reduction of volume does not exist, and therefore a decrease in viscosity is not seen when such morphologies are created. Coupled with knowledge from μDSC integration, at this temperature there is still much ordering left to take place before the transition has ended – only 41% of the transition has occurred by the viscosity peak temperature for Sigma agar and, for Luxara with no comparable viscosity peak, at the same temperature almost 60% of the transition is complete. Concurrent analysis of thermal reaction kinetics and rheological properties provides some insight, but the origin of such differences in particle morphology is not clear and should be subject to further exploration.

**Fig. 5:**
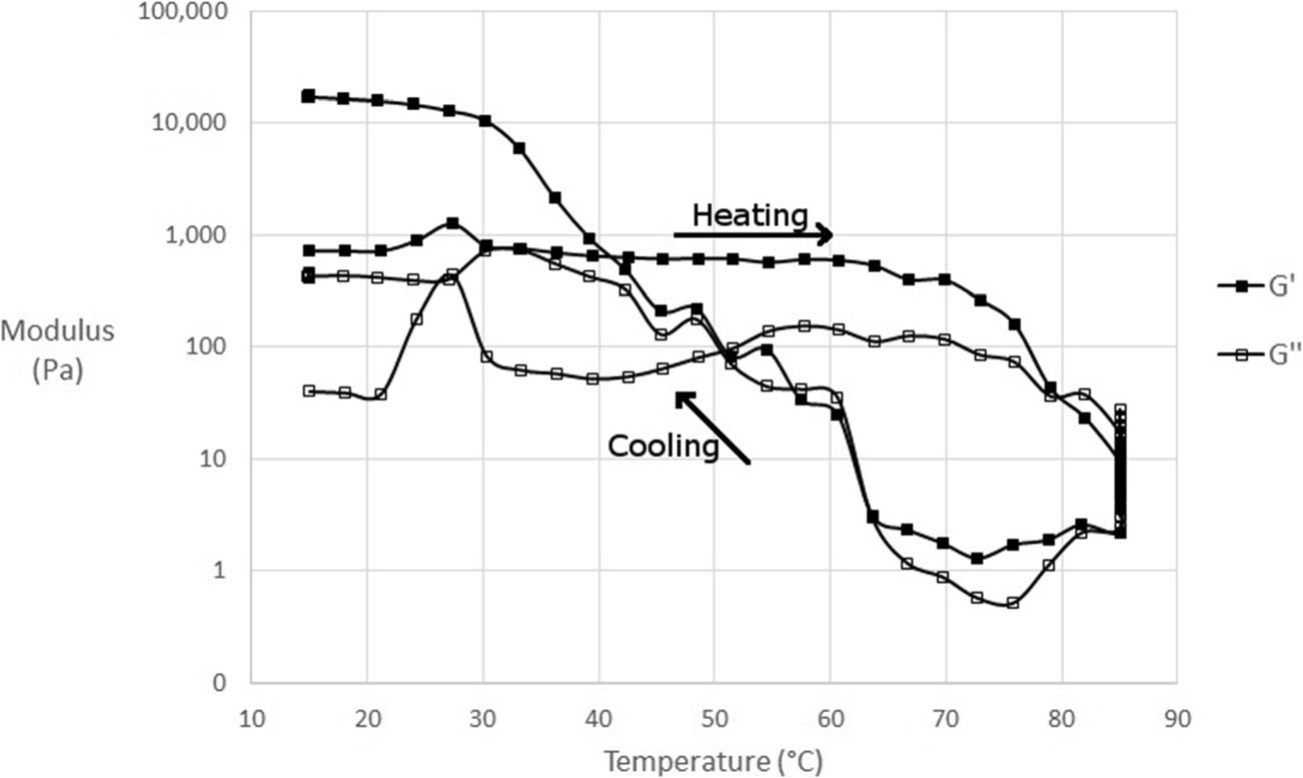
G’ and G” progression of 2% Luxara agar fluid gel over a temperature sweep from 15 to 85 °C and back down, with a five minute isothermal hold at 85 °C

As mentioned in the materials and methods amplitude sweeps of fluid gels produced were conducted to determine the linear viscoelastic region. Whilst gently oscillating within this region, to monitor viscoelastic moduli, the fluid gels were heated from 10 °C to a value between 55 and 85 °C, held at this point for five minutes then cooled to 10 °C. Figure 5 shows an example of a *G’* and *G”* trace versus temperature obtained from one of these sweeps when the fluid gel was heated to 85 °C after production. At temperatures lower than those attributed to the melting endotherm in μDSC the *G’* stays relatively constant but begins to decrease rapidly as molecular mobility increases and agar fluid gel particles begin to destabilise and undergo helix to coil transition due to sufficient thermal energy input. The *G’* remains low at these elevated temperatures then begins to rise again on cooling at temperatures associated with the μDSC gelation event. Further cooling beyond this point back to 10 °C results in a final *G’* much larger than the initial fluid gel sample, closer to that of the quiescent gel. Heating to 85 °C was the highest temperature sweep conducted for all fluid gel samples and was attributed to the highest final *G’* values for both types of agar at each concentration. The exception to this is 2% Luxara fluid gels made in the rheometer, where 95–100 °C heating steps were conducted and are presented with μDSC data in Fig. 7. The *G’* after this temperature sweep was consistent with measurements for quiescent gels. This trend was also seen when samples created in the stirred pot were loaded into the rheometer and subjected to identical temperature sweeps.

**Fig. 6:**
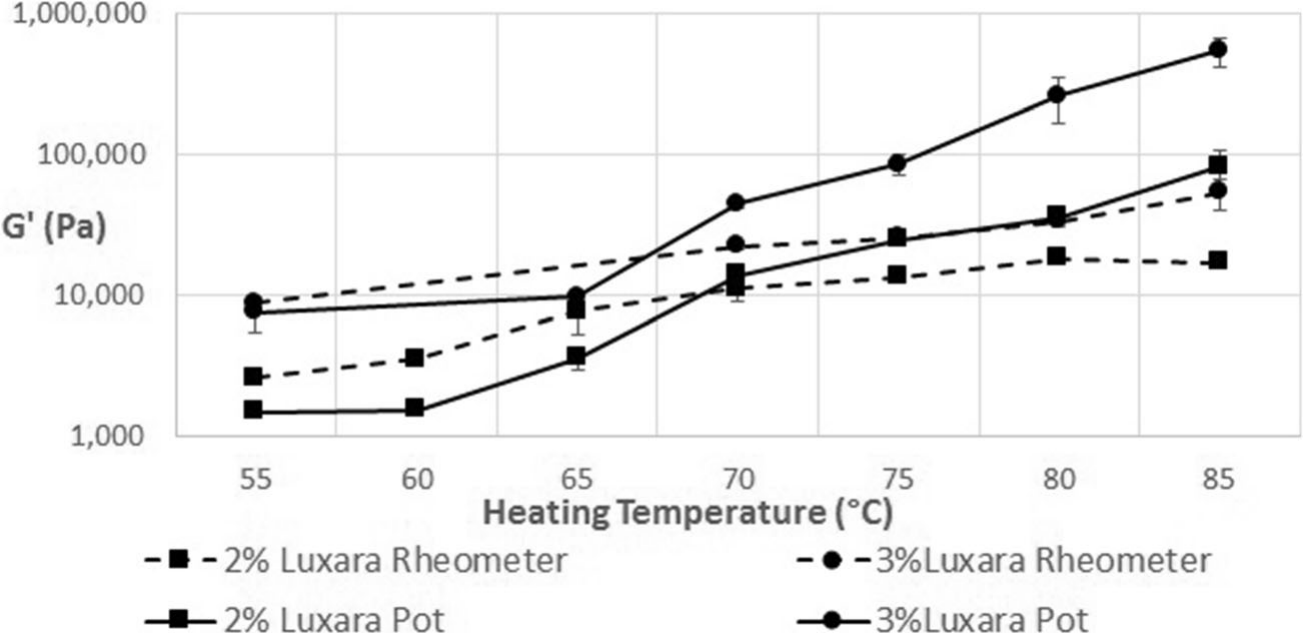
Final G’ of thermally cycled fluid gels plotted against the temperature they were heated to

**Fig. 7:**
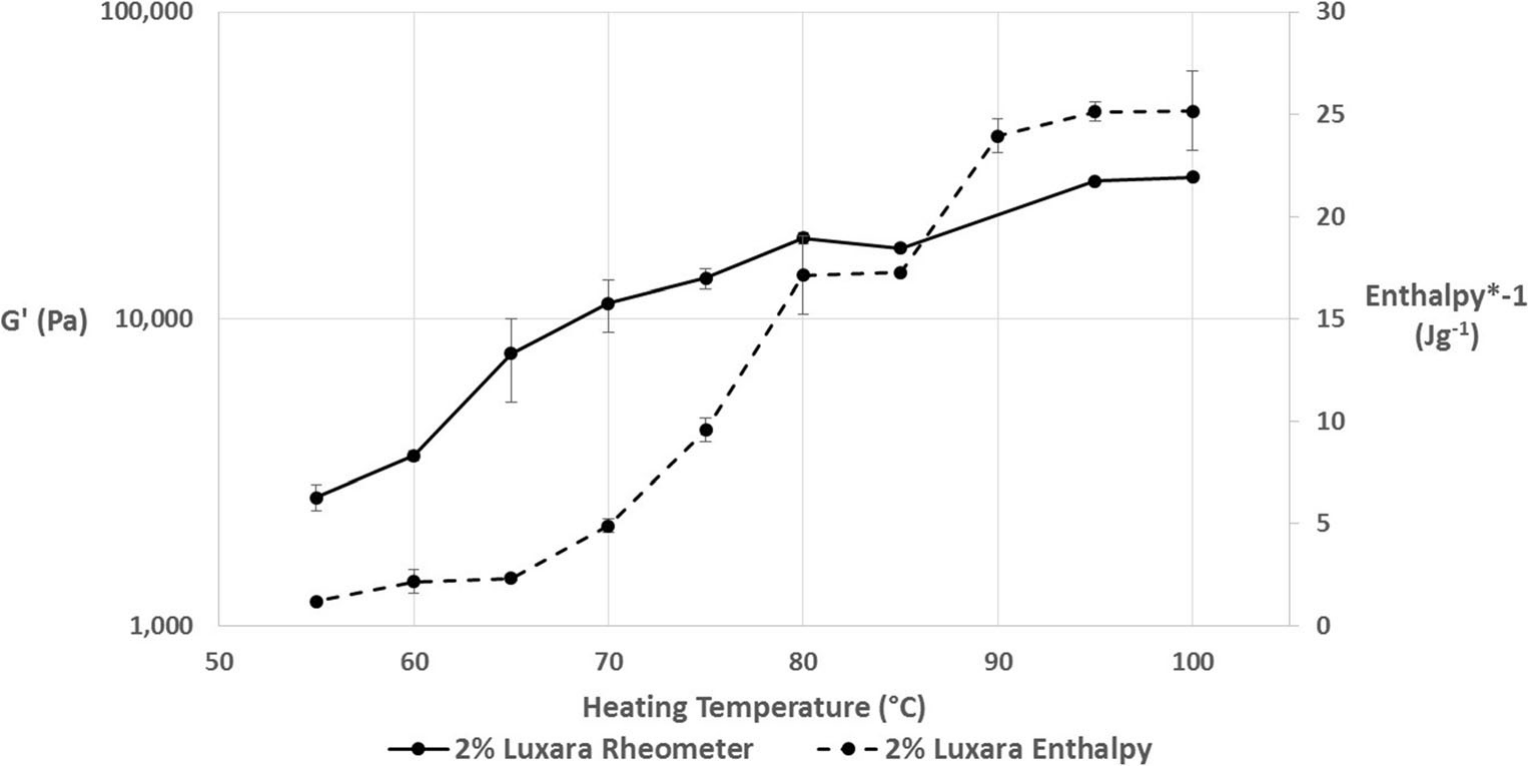
A plot of final G’ values of a 2% Luxara fluid gel versus exotherm enthalpy on heating to various temperatures

Figure 6 plots the end *G’* once re-cooled versus heating temperature for 2 and 3% fluid gels of Luxara agar made both by the stirred pot and rheometer methods. The final *G’* achieved between heating to 55 °C versus 85 °C is an increase of almost 2 log for samples made in a stirred pot and 1 log for those made in the rheometer. The higher the heating temperature the more disordering of aggregates and helix to coil transition occurs. In this method of stopping the heating process at a point along the melting endotherm, isothermally holding and then cooling there will be different extents of particle dissolution. At lower temperatures, some aggregates will break and smaller, kinetically trapped helices or chains which may be in the ‘hairy’ regions protruding into the continuous phase can be more easily solubilised than those at the core of the gel particle. This is likely to be due to the formation of tails being at a lower temperature, as discussed earlier, thus they would have a lower melting temperature compared with the particle core. Therefore on cooling the continuous phase is likely to be a weak gel with reinforcing dispersed gelled particles remaining in it. Conversely at higher temperatures most of the aggregates will disorder, enabling double helices to be solubilised in the coil state, thus the continuous phase will have a relatively higher concentration of agar able to take part in gelling but few reinforcing particles. The effect of particle reinforcement may be more pronounced in those fluid gels made via the stirred pot method due to a larger starting particle size, explaining the higher *G’* values observed when heating above 70 °C. All temperatures presented in Fig. 7 were below the agar melting endset, determined by μDSC, therefore even the final *G’* of samples heated to 85 °C was lower than that for quiescently set agar. Additionally, a five minute isothermal hold is insufficient to allow for full solubilisation of agar molecules but is more representative when considering potential mechanisms in the 3D printing application.

In Fig. 7, data from Fig. 3 and Fig. 6 is combined to show a trend which exists dependent on the temperature a fluid gel is heated to and subsequently cooled. As previously mentioned the peak maximum of the melting endotherm for a Luxara fluid gel was calculated as 76.8 ± 0.9 °C. The enthalpy of gelation after heating to either side of this (i.e. 70 °C or 80 °C) increases dramatically over this range, after which a plateau is observed for heating to 80 °C and 85 °C, before a further increase and levelling out at 90–100 °C. This is to do with the melting endotherm endset occurring just after 90 °C (see Fig. 2). It is interesting that this plateau region when heating to 80–85 °C also exists when considering the *G’* achieved on cooling after a temperature sweep. Within this temperature range, after the melting peak maximum, there could be constrained reordering within the fluid gel particle. This would cause a plateau region for both enthalpy on gelation and the measured *G’*, but after this reordering and heating to temperatures beyond 85 °C melting is able to be completed. At 80 °C 72.5% of the endothermic reaction has taken place with a further 20% occurring between 80 and 85 °C, based on integration of the endothermic transition peak, yet re-heats to these temperatures result in plateau values for *G’* and exotherm enthalpy on cooling. Therefore this region begins to indicate heterogeneity between fluid gel particle core and surface upon formation, as there is clearly a significant amount of ordering occurring based on the endotherm integration data, but no uniform increase in gel strength, based on rheology and gelation enthalpy. A further rise in both sets of data upon re-heats to temperatures above 85 °C indicates that this heterogeneity has been overcome, and ordering may continue in a more similar manner to a quiescently set gel. This is exhibited by a *G’* consistent with quiescent gel measurements observed upon conducting temperature sweeps to 95 °C and 100 °C and determination of 99.9% of the endothermic reaction being complete at 90 °C for Luxara agar, based on integration.

Results presented in the sections above can allow for the understanding and development of a model based on heated and re-set thermoreversible fluid gels to be used in practical application. This has been demonstrated for 3D printing using high viscosity ink jet.

### 3D Printing Fluid Gels

The main parameters altered to attain good jetting of droplets with the Nordson PicoPulse valve and Pico Touch controlling system were the supplied pressure, pulse (time the valve remains open), close voltage, stroke percentage and valve open and close times within a cycle time of 10 ms. Preliminary trials have identified suitable operating parameters for fluid gel samples at ambient temperature. For a 2% Sigma Agar fluid gel parameters used were; 3 bar pressure, 0.3 ms pulse, 120 V, 50% stroke, 0.25 ms opening time and 0.2 ms closing time. As heat was applied at the nozzle the solution viscosity decreased therefore pressure was decreased to 2 bar and small adjustments were made to other parameters depending on the printing definition achieved at any given temperature.

**Fig. 8:**
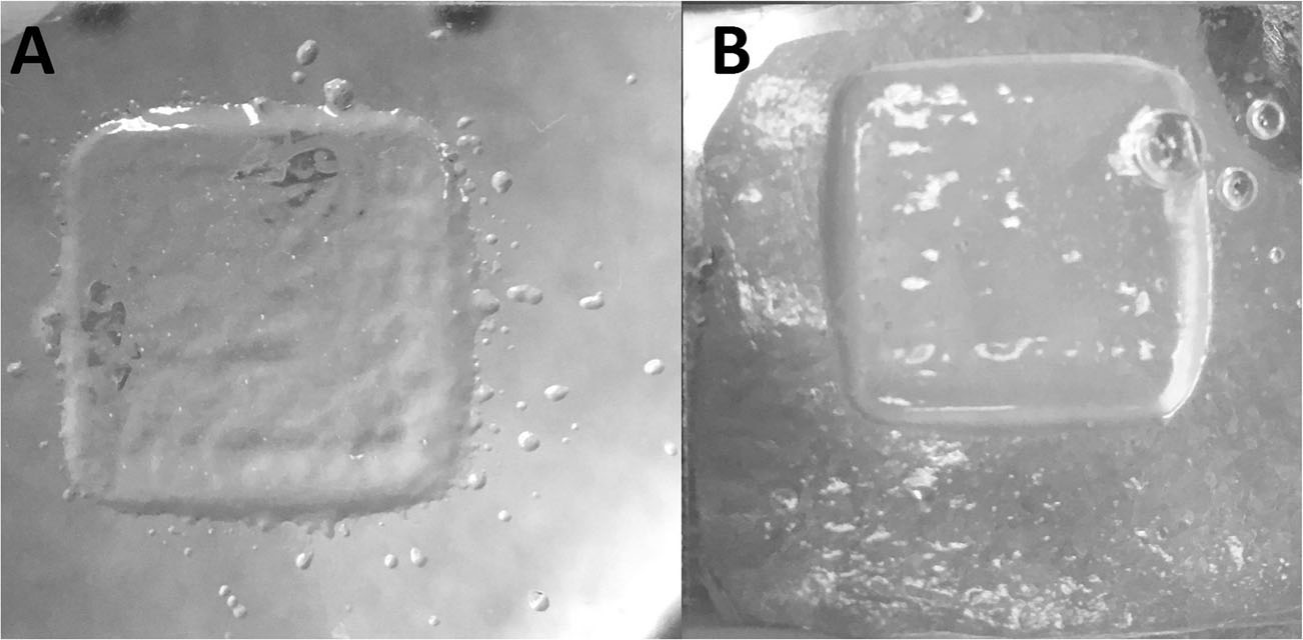
20 layers of 2% Sigma fluid gel printed with 1000 μm drop spacing on glass (**a**) and agar (**b**) coated substrates

Printing was initially conducted onto a glass microscope slide however it was discovered that better printed droplet resolution, shape retention and structure removal could be achieved if the glass slide substrates were coated with a thin layer of quiescently set agar, illustrated in Fig. 8. This was of particular importance when printing at elevated temperatures, or when using the substrate cooling stage.

**Fig. 9:**
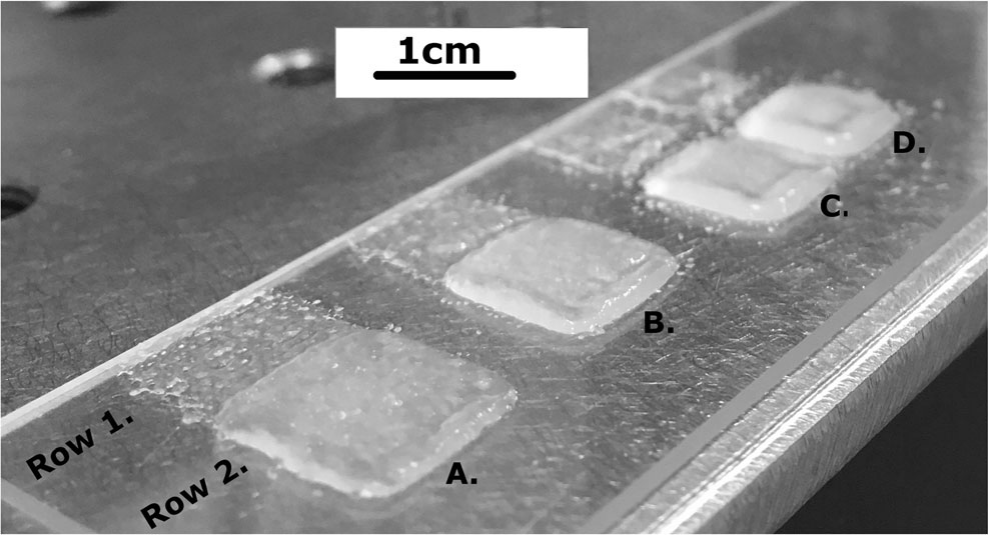
Luxara fluid gel printed at 700 (A.), 600 (B.), 500 (C.) and 400um (D.) in 1 (Row 1.) and 5 (Row 2.) layers

During printing at a variety of temperatures it was possible to create structures of variable height without detriment to the layer underneath. Figure 9 shows 1 layer versus 5 layers (rows 1 and 2) of varying droplet spacing 700 μm, 600 μm, 500 μm, 400 μm (A-D) of Luxara agar fluid gel. When printing the fluid gel at ambient nozzle temperature onto an ambient substrate the structure held form and could be removed on the quiescently set agar layer, however it was difficult to separate the fluid gel structure itself from the quiescent agar layer whilst maintaining structural integrity. In this instance it was found that using a cooled substrate (below 0 °C) allowed better layer cohesion of printed fluid gel particles, likely by promoting a physical association through squeezing out continuous phase liquid that the particles were suspended in through freezing and thawing (akin to the freezing step used in agar production to purify extracts [22, 33]). When printing at elevated temperatures e.g. 70 °C the print rate (100 Hz) was sufficient to enable partial cooling of one layer before the next was deposited on top to hold its shape under the weight of the next layer but not so much that it had fully gelled and the layers did not adhere to one another in the z direction. This is aided by the comparatively lower volume, dropwise manner of depositing the fluid gel layers in this particular system rather than in other methods of hydrocolloid 3D printing such as extrusion where deposition is via a continuous stream.

**Fig. 10:**
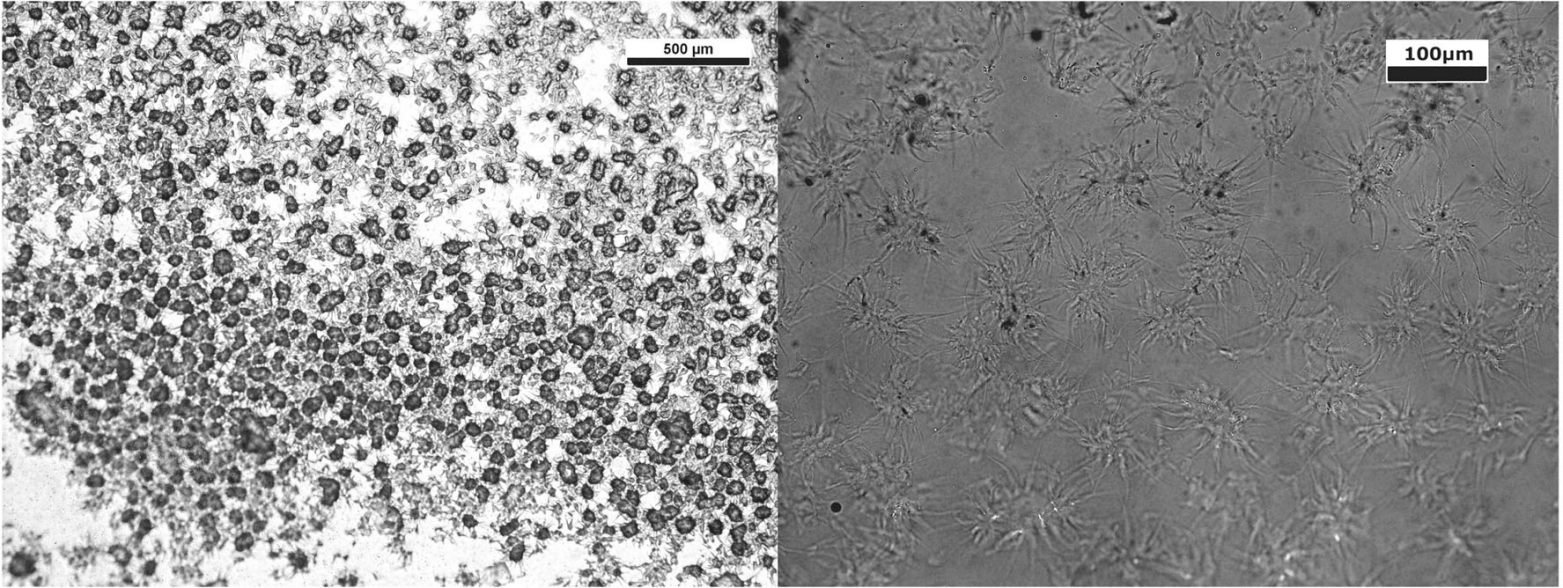
Ambient temperature printing of agar fluid gels with particles distinctly visible at ×5 and ×20 magnifications

Light microscopy was used to observe fluid gel droplet arrays straight after printing to prevent the samples drying out. Figure 10 shows light micrographs of printed fluid gel at ambient nozzle and substrate temperatures using ×5 (left) and ×20 (right) lenses. The individual fluid gel particles are clearly visible and their morphology at higher magnification mirrors phase contrast results after production (c.f. Fig. 1). As the printing nozzle temperature was increased there were fewer and fewer fluid gel particles visible within individual droplets and they more closely resembled a gel network, as expected from rheological and thermal analysis findings.

## Conclusions and Future Work

We have demonstrated the feasibility and benefit of using fluid gels as a feedstock for 3D printing with application in a high viscosity jetting system. When printing thermoreversible gels the creation of a fluid gel from the polymer of interest prior to printing negates the requirement of keeping the feedstock at elevated temperatures, as has been necessary in previous studies. As demonstrated elsewhere in the literature fluid gel production is not restricted to thermally gelling polymers, it could be useful in the future to look into printing with alternative fluid gels depending on the application. For agar fluid gels there is a clear link between the dependence of particle size, shape and rheological properties on the type of agar used, method of production and concentration. Thermal and rheological studies were concordant in showing a tuneable gelation event after applying a heating and cooling cycle to fluid gels with the extent of gelation dependent on the temperature heated to. The combination of these studies led to the conclusion that the temperature region between 80 and 85 °C, when considering the melting of agar fluid gels, can be attributed to constrained reordering within the particle, determined by a plateau of gelation enthalpy and final *G’* after sweeps to and from these temperatures. It was this knowledge which enabled the application of thermoreversible agar fluid gels to high viscosity jetting, creating structures in 3D with varied hardness as a result of the extent of melting of fluid gel particles and forming a fully quiescent set gel or a particle reinforced gel, by controlling the nozzle temperature. When printing fluid gels under ambient conditions, the use of a freezing substrate can cause concentration of agar fluid gel particles and expulsion of some bound water so that they pack closely together to form a structure without the application of heat. This phenomenon should be explored further in addition to conducting more characterisation of all printed structures with the aim of printing more complex geometries. This may also be made possible by printing with ‘mixed’ fluid gels, where relative particle numbers will be determined by both formulation and processing conditions. This addition will allow for further structure control and multiple nozzles, printing different fluid gel feedstocks which provides an exciting future, whose limits are constrained by imagination.
